# Isolation, Identification and Characterization of Endophytic Bacterium *Rhizobium oryzihabitans* sp. nov., from Rice Root with Biotechnological Potential in Agriculture

**DOI:** 10.3390/microorganisms8040608

**Published:** 2020-04-22

**Authors:** Juanjuan Zhao, Xia Zhao, Junru Wang, Qi Gong, Xiaoxia Zhang, Guishan Zhang

**Affiliations:** 1 Key Laboratory of Microbial Resources Collection and Preservation, Ministry of Agriculture, Institute of Agricultural Resources and Regional Planning, Chinese Academy of Agricultural Sciences, Beijing 100081, China; zhaojuanjuan3867@163.com (J.Z.); junru1120102514@gmail.com (J.W.); gongqi_97@163.com (Q.G.); zhangxiaoxia@caas.cn (X.Z.); 2 Beijing Research Institute of Chemical Engineering and Metallurgy, Beijing 101149, China; 18804894622@163.com

**Keywords:** Plant-promoting endophytic bacteria, *Rhizobium oryzihabitans* sp. nov., Comparative genome analysis

## Abstract

A flagellate, rod–shaped bacterium designated strain M15^T^ was isolated from rice roots. Phylogenetic analysis based on the sequences of the 16S rRNA, housekeeping genes and genomes showed that the isolate belonged to the genus *Rhizobium*, with the highest 16S rRNA similarity to *Rhizobium radiobacter* LMG140^T^ (99.64%) and *Rhizobium pusense* NRCPB10^T^ (99.36%), respectively. The complete genome of the strain M15^T^ has a 59.28% G+C content, and the highest average nucleotide identity (ANI) and DNA-DNA relatedness (DDH) values were obtained with *R. radiobacter* LMG140^T^ (88.11%, 54.80%), *R. pusense* NRCPB10^T^ (86.00%, 53.00%) and *R. nepotum* 39/7^T^ (88.80%, 49.80%), respectively. Plant growth-promoting characteristics tests showed that the strain M15^T^ produced siderophore, 1–aminocyclopropane–1–carboxylate (ACC) deaminase and indole-3-acetic acid (IAA) and also produced some secondary metabolites according to the analysis of the comparative genomes. Based on the data mentioned above, we proposed that the strain M15^T^ represented a novel species of the genus *Rhizobium*, named *Rhizobium oryzihabitans* sp. nov. The type strain is M15^T^ (=JCM 32903^T^  = ACCC 60121^T^), and the strain M15^T^ can be a novel biofertilizer *Rhizobium* to reduce the use of synthetic fertilizers for plant growth promotion.

## 1. Introduction

The genus *Rhizobium*, belonging to the family Rhizobiaceae within the order Rhizobiales of the class Alphaproteobacteria, was first described by Frank as root and/or stem nodulating bacteria [1]. Generally, most of the *Rhizobium* species have been isolated from the nodules on *leguminous* plants with the function of symbiotic nitrogen-fixing. However, some free-living *Rhizobium* strains have frequently been isolated from soil [2,3,4,5], water [6,7], rhizosphere and plant roots [8]. In addition, several *Rhizobium* species have been described on the basis of non-symbiotic strains isolated from rice recently, such as *Rhizobium oryziradicis*, *Rhizobium rhizospherae*, *Rhizobium pseudoryzae*, *Rhizobium rhizoryzae*, and *Rhizobium oryzicola* [9,10,11,12].

Plant growth-promoting rhizobacteria (PGPR) is a group of rhizosphere bacteria that can enhance plant growth by phosphate solubilization, siderophore production, biological nitrogen fixation, production of 1–aminocyclopropane–1–carboxylate (ACC) deaminase, and production of indole-3-acetic acid (IAA) [13]. *Rhizobium* is a well–known plant microbiota for nitrogen fixation traits with *legume* host and non-*legume* plant growth promoters [14,15,16]. Such as, *Rhizobium leguminosarum* trifolii E11 is able to promote rice plant growth by phosphate solubilization and IAA production; *Bradyrhizobium japonicum* can promote the growth of radishes by siderophore production [16], and so on. The mechanisms have been studied thoroughly and used widely in agricultural applications. 

In this study, we reported a novel species of *Rhizobium* isolated from rice roots during the studies of bacterial diversity. The aims of this work were: 1) To isolate and identify the novel *Rhizobium* strains; 2) To characterize the plant growth-promoting traits of the *Rhizobium* strains, such as siderophore, ACC deaminase, IAA production, rice plant growth enhancement, and 3) To analyze the genomes and secondary metabolites of the *Rhizobium* strains against closely related species, and test their possible application as biofertilizers.

## 2. Materials and Methods 

### 2.1. Strain and Culture Conditions

The endophytic strain M15^T^ was isolated from surface-sterilized rice roots collected from Beijing, China. The isolation and purification were performed on yeast extract mannitol (YMA) medium [17] at 30 ℃. Strains were preserved as glycerol suspension (20%, v/v) at –80 ℃ and at –4 ℃ in freeze-drying ampoules for further characterization.

To study the chemotaxonomic and molecular characteristic, biomass was collected from YMA medium at 30 ℃ for 2 days. The reference strain *R. radiobacter* LMG140^T^ and *R. pusense* NRCPB10^T^ were obtained from Deutsche Sammlung von Mikroorganismen und Zellkulturen GmbH (DSMZ; Germany). Two reference strains were cultured under the same conditions as strain M15^T^ for comparative analysis.

### 2.2. Morphological, Physiological and Biochemical Characteristics

Cell morphology was examined using a light microscope (CX21; Olympus). Gram-staining was carried out using the Gram-Stain Kit (solarbio, Beijng, China). Growth features were tested in different temperatures at 4, 15, 25, 30, 37, 40, 45, 50 ℃ and pH 3.0–12.0 (at 1.0 unit intervals) on YMA medium. Salt tolerance was tested on YMA with NaCl concentrations of 0, 1.0, 3.0, 5.0, 7.0, 10.0% (w/v). Motility was observed by semisolid culture–medium (0.4% agar added). Flagellum was observed by transmission electron microscopes (JEM-1400, Japan). Oxidase and catalase activities were determined by using 1% (w/v) tetramethyl–p–phenylenediamine and 3% (v/v) H_2_O_2_, respectively. The basic biochemical characteristics were investigated on Biolog GN2 Microplates (Hay-ward, CA, USA) and API–20NE test strips (bioMérieux, Marcy-I’Etoile, France).

### 2.3. Molecular Studies

Genomic DNA was extracted from pure cultures using Bacteria DNA Kit (Axygen Scientific, Union City, CA) according to the manufacture’s protocol. The 16S rRNA gene was amplified using the universal primers 27F and 1492R according to Lane et al. [18]. The housekeeping genes *recA*, *ropB*, *atpD* were amplified using the methods of Martens et al. [19,20]. The 16S rRNA gene sequences similarity and multiple sequences alignment were analysed using EzTaxon–e Service [21], CLUSTALW [22], respectively. Similarities of housekeeping genes were performed by the National Center for Biotechnology Information Search database BLAST program and multiple sequences alignment was carried out by CLUSTALW. Phylogenetic trees were constructed using MEGA 7.0 software with neighbor–joining methods [23,24,25]. 

Genome sequencing was carried out using Illumina next–generation sequencing combined with PacBio single–molecule long–read sequencing technology. Software A5–miseq [26], SPAdes [27], HGAP4 [28] and CNAU (V1.6) [29] were used for genome assembly. Prokaryotic Genome Annotation Pipeline (PGAP) was used for genome annotation by NCBI. The genome sequences of strain M15^T^ was submitted to NCBI and the accession number is SAMN14048699. The average nucleotide identity (ANI) values were calculated using ANI Calculator in the EZBioCloud. The DNA–DNA hybridization (DDH) estimates were using GGDC (Genome-to-Genome Distance Calculator) with the BLAST+ (recommended) method [30]. 

### 2.4. Fatty Acid Analysis

For cellular fatty acids analysis, strains M15^T^, *R. radiobacter* LMG140^T^ and *R. pusense* NRCPB10^T^ were grown on YMA for 2 days at 30 ℃. Cultures were harvested and fatty acid methyl esters were prepared and separated using methods described by Sasser [31] and were identified with the MIDI Sherlock Microbial Identification system.

### 2.5. Analysis of Plant Growth-Promoting Characteristics

Production of IAA was analysed according to Glickmann and Dessaux [32]. Quantitative analysis was performed at 50 μg/ml concentrations of tryptophan. A standard graph of IAA in the range of 0–250 μg/ml was used to measure the concentration of IAA produced by strain M15^T^. Phosphate solubilization was measured on Pikovaskaia’s (PKO) inorganic phosphorus medium at 30 ℃ and observed daily for up to 7 days. The presence of a transparent halo around the colonies demonstrated ability of phosphate solubilization. Siderophore production was detected on chrome azurol S (CAS) agar plates, the color change around the colonies from blue to orange indicated the presence of siderophores [33]. ACC deaminase activity was assayed according to a method described by Perone and Glick [34] with modification. Effects of strain M15^T^ on rice growth was evaluated under liquid culture condition formulated with conventional nutrient composition from the International Rice Research Institute according to Habibi et al. [35] with modification. The high–quality disease–free rice seeds were surface-sterilized. Germinated seeds were growth in pots for 12 days and then transplanted into liquid culture medium inoculated with 5 mL bacterial culture at a cell density of 10^9^ CFU/mL. Uninoculated plants were used as a control. The length and fresh weights of roots and shoots were recorded. All of the experiments were performed in triplicate. The significance of differences between treatments and controls was analyzed using Tukey’s test (*P* < 0.05). 

### 2.6. Comparative Genomics of Rhizobium Species

Twelve close bacteria belonging to the genus *Rhizobium* were selected for comparative studies. The 12 bacteria were as follows: *Rhizobium nepotum* 39/7^T^, *Rhizobium radiobacter* LMG140^T^, *Rhizobium pusense* NRCPB10^T^, *Rhizobium hainanense* CCBAU57015^T^, *Rhizobium freirei* PRF81^T^, *Rhizobium miluonense* HAMBI2971^T^, *Rhizobium loessense* CGMCC1.3401^T^, *Rhizobium mongolense* USDA1844^T^, *Rhizobium tibeticum* CGMCC1.7071^T^, *Rhizobium grahamii* CCGE502^T^, *Rhizobium altiplani* BR10423^T^ and *Rhizobium oryzihabitans* M15^T^. The Insert Genome Into Species Tree 2.1.10 in KBase was used to constructed a species tree [36]. The phylogenetic tree based on orthologous proteins of the *Rhizobium* genus was constructed by FastTree [37]. Pan genome analyses were performed by Build Pangenome with OrthoMCL-v2.0 in KBase [36]. A clustering tool MCL was used to cluster protein families. A 50% sequence identity was considered as the cut-off value for the orthologous clustering to obtain the pan and core genome. The virulence factors analysis of strain M15^T^ and *R. radiobacter* LMG140^T^, a well known pathogenic species to cause the crown gall disease across thousands of different plant species [38], were performed using Virulence Factors of Pathogenic Bacteria. The secondary metabolite biosynthetic gene clusters of the 12 *Rhizobium* species were predicted using antiSMASH 5.0 [39]. The synteny maps of the gene clusters were generated using Easyfig [40].

## 3. Results and Discussion

### 3.1. Morphological, Physiological and Biochemical Characteristic

Strains M15^T^ was rod and aerobic, flagellate (Supplementary Figure S1), Gram–staining–negative bacteria. Colonies were circular and pearl white on YMA medium at 30 ℃. Growth occurred at 15–50 ℃ (optimal 30 ℃).The pH range for growth was 5.0–12.0 and the tolerance of NaCl was up to 7.0% (w/v), which indicated that strain M15^T^ endowed with inherent capability to cope with high concentration of salt or overly alkaline in soil. Thus, it has the potential for applicaiton in the form of bioinoculants to make the survival of plants easier under extreme saline or alkaline conditions. Strain M15^T^ was observed to be catalase- and oxidase-positive. Other physiological and biochemical characteristics of the novel isolate and reference strains were depicted in Table 1. Strain M15^T^ could be distinguished from *R. radiobacter* LMG140^T^ in utilization of D-psicose, methyl pyruvate, mono–methyl–succinate, *β*–hydroxy butyric acid and growth at NaCl (7.0%) and pH11.0. It differed from *R. pusense* NRCPB10^T^ in assimilation of *N*–acetyl–D–galactosamine, D–psicose, methyl pyruvate, mono–methy–succinate, formic acid, *β*–hydroxy butyric acid, *α*–keto glutaric acid, L–threonine, inosine, uridine, 2,3–butanediol. Discriminative features between the novel isolate and its close relatives are detailed in Table 1.

### 3.2. Molecular Studies

The genome of strain M15^T^ consists of a circular chromosome of 3,195,345 base pairs, a linearized chromosome of 1,948,381 base pairs and seven plasmids. It was predicted to contain 5670 genes, including 5110 protein-encoding genes, 16 rRNA genes, 59 tRNA genes, 4 ncRNA genes and 483 pseudo genes. The DNA G+C content of strain M15^T^ was 59.28 mol %, which was within the range reported for *Rhizobium* species (57–66 mol %) [41], implying that it belong to the genus *Rhizobium*. The ANI between strain M15^T^ and *R. nepotum* 39/7^T^, *R. radiobacter* LMG140^T^, and *R. pusense* NRCPB10^T^ were 88.80%, 88.11%, 86.00%, respectively, which is below the referral threshold value (95–96%) for species demarcation suggested by Kim et al. [42] and Richter et al. [43] (Supplementary Table S1).

The DDH between strain M15^T^ and *R. radiobacter* LMG140^T^, *R. pusense* NRCPB10^T^ were 54.80%, 53.00%, respectively, which is lower than the same species threshold of 70% [44,45] (Supplementary Table S2). These results showed that strain M15^T^ represent a novel species of the genus *Rhizobium*.

**Figure 1:**
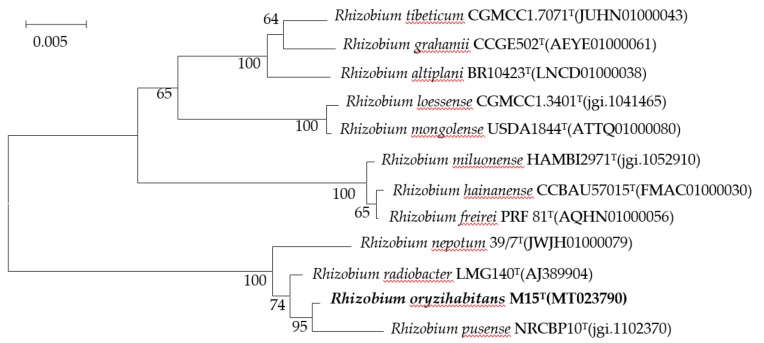
Phylogenetic tree generated with the neighbor-joining algorithm based on 16S rRNA gene sequences showing the phylogenetic positions of strain M15^T^ and related taxa. Bootstrap values with more than 50% are shown on the nodes as percentages of 1000 replicates. *Rhizobium grahamii* CCGE502^T^ (AEYE01000061) was used as an outgroup. The scale bar equals 0.005 change per nucleotide position.

The almost complete 16S rRNA gene sequences of strain M15^T^ was determined and subjected to phylogenetic analysis. Similarity search in EzTaxon–e revealed that strain M15^T^ was most closely related to the members of the genus *Rhizobium*, and showing the highest 16S rRNA gene sequence similarity to *R. radiobacter* LMG140^T^ (99.64%) and *R. pusense* NRCPB10^T^ (99.36%). A phylogenetic tree was constructed with the Neighbour–joining (NJ) method using MEGA 7.0 (Figure 1), which indicating the isolate was clustered into a novel branch within the genus *Rhizobium*.

To further explore the phylogenetic relationships between strain M15^T^ and the closely related *Rhizobium*, the partial *recA* (496 bp), *ropB* (961 bp) and *atpD* (489 bp) gene sequences were also detected. The strain M15^T^ shared the highest *recA* (91.94%), *ropB* (94.81%)and *atpD* (97.05%) gene sequences similarity with closely related *Rhizobium* species, so the high similarity of the housekeeping gene sequences, and their phylogenetic trees between strain M15^T^ and closely related strains of the genus *Rhizobium* (Supplementary Figure S2), also indicating that strain M15^T^ represent a novel species within the genus *Rhizobium*.

### 3.3. Cellular Fatty Acid Composition 

The principal cellular fatty acids of strain M15^T^ are Summed Feature 8 (C_18:1_*ω7c* and/or C_18:1_*ω6c*) and Summed feature 2 (aldehyde–C_12:0_ and/or unknown equivalent chain length), which are the characteristic compositions of root-nodule bacteria [46]. However, the novel isolate could be divided from related species mainly by possessing C_9:0_ and distinctly different content of the C_19:0_ cyclo*ω8c*. The details of the fatty acid profiles of strain M15^T^ and reference strains are shown in Table 2. 

### 3.4. Analysis of Plant Growth-Promoting Characteristic

To determine the properties of plant growth-promoting of strain M15^T^, several related functions were characterized. The results showed that strain M15^T^ produced IAA with 85.32 μg per ml under the OD600 nm of 1.0. IAA is the main auxin in plants, regulating growth and developmental processes such as cell division and elongation, tissue differentiation, apical dominance, and responses to light, gravity, and pathogens [47]. After growth in CAS medium, we observed an orange halo surrounded by the colony indicative of siderophore production. Siderophores are low molecular-weight iron chelators that directly promote plant growth by converting unavailable ferric oxides into absorbable forms for plants or indirectly by binding to the available forms of iron in soil and depriving pathogens in the vicinity of iron [35,48]. The strain M15^T^ as IAA and siderophore-producing bacteria, has the potential to be developed into bio–fertilizer to enhance plant growth and induce plant resistence to pathogens.

**Figure 2:**
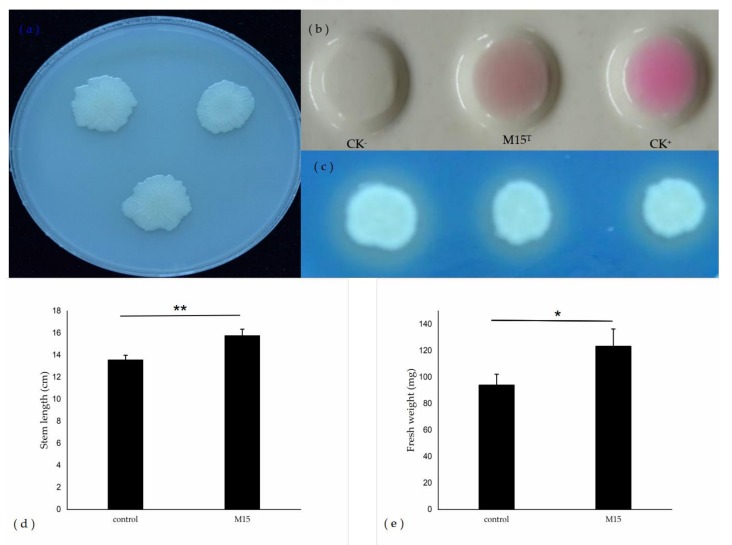
(**a**) Phosphate solubilization test in PKO medium; (**b**) detection of indole acetic acid production; CK^−^ indicates the negative control using non-inoculated medium mixed with an equal volume of colorimetric; CK^+^ indicates the positive control using 100 μg/ml indole-3-acetic acid (IAA) standard mixed with an equal volume of colorimetric; (**c**) siderophore production with three repetitions; (**d,e**) Effects of strain M15^T^ on rice plant growth; d, e, indicate the effects of strain M15^T^ on rice stem length and fresh weight (total weight of roots and stems), respectively. ** value is significantly different from the control, within each column (P < 0.01); * value is significantly different from the control, within each column (P < 0.05); Each treatment have at least three biological replicates.

Strain M15^T^ is able to produce ACC deaminase with the enzyme activity of 240nmol α-ketobutyrate/h·mg (Figure 2), which is high than the ACC deaminase activity level, namely approximately 20 nmol α–ketobutyrate/h·mg, to act as a PGPR [34]. Ethylene is a gaseous phytohormone, which is involved in a broad spectrum of the plant life cycle, including seed germination, nodulation, flower senescence, fruitripening, and leaf abscission [49]. High concentration of ethylene lead to growth inhibition, chlorosis, or even death of plant [49]. ACC deaminase can modulate the level of ethylene to facilitate plant resistence under different environmental stresses, especially waterlogged conditions, and contribute to plant growth and development [49,50]. Therefore, ACC deaminase–producing bacteria, like strain M15^T^, can be developed into inoculants to be used as alternatives to various agrochemicals.

The results of seed inoculation assays showed that the strain M15^T^ promoted the growth of rice compared to the controls. The length of stem and fresh weight of the inoculated seedlings was significantly increased with respect to the uninoculated seedlings. These results showed that the strain M15^T^ promoted plant growth by affecting the development of the stem and the fresh weight of rice root.

### 3.5. The Comparative Genome Analyses from 12 Closely Related Strains of the Genus Rhizobium

**Figure 3:**
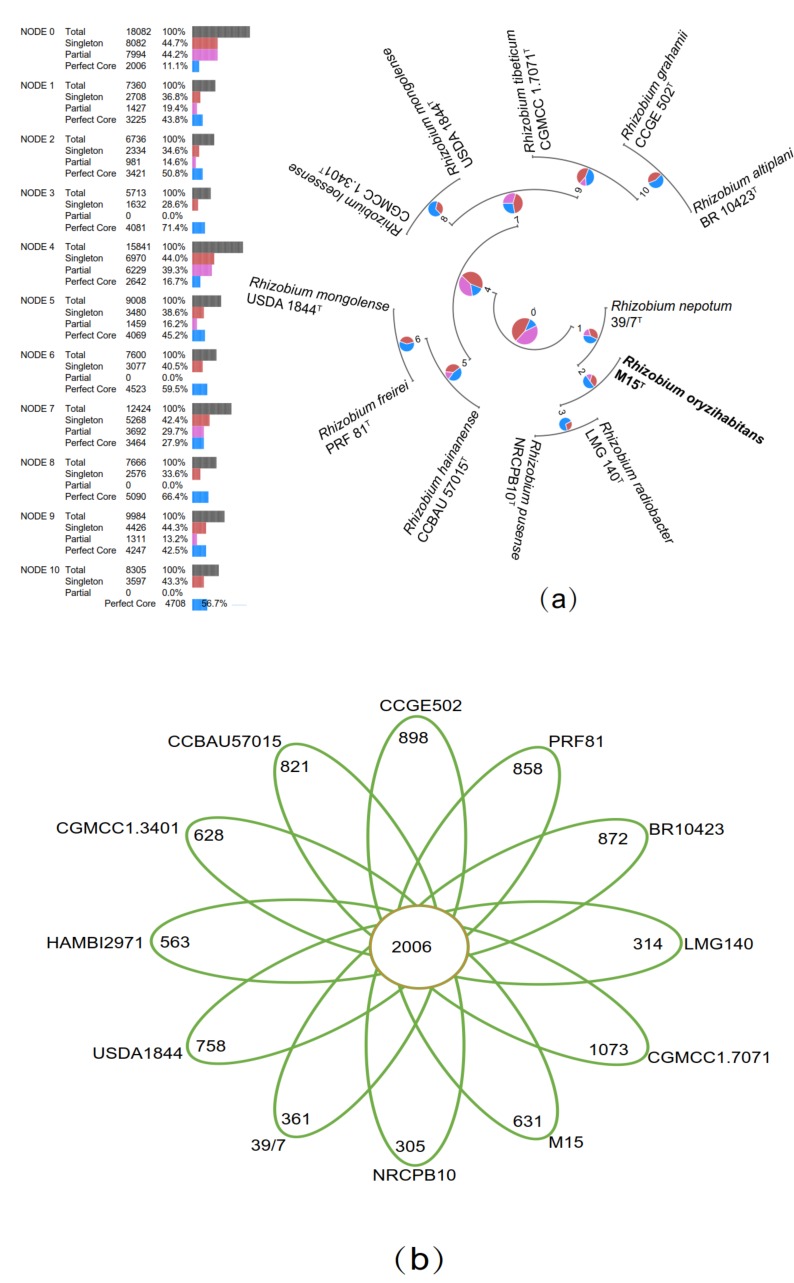

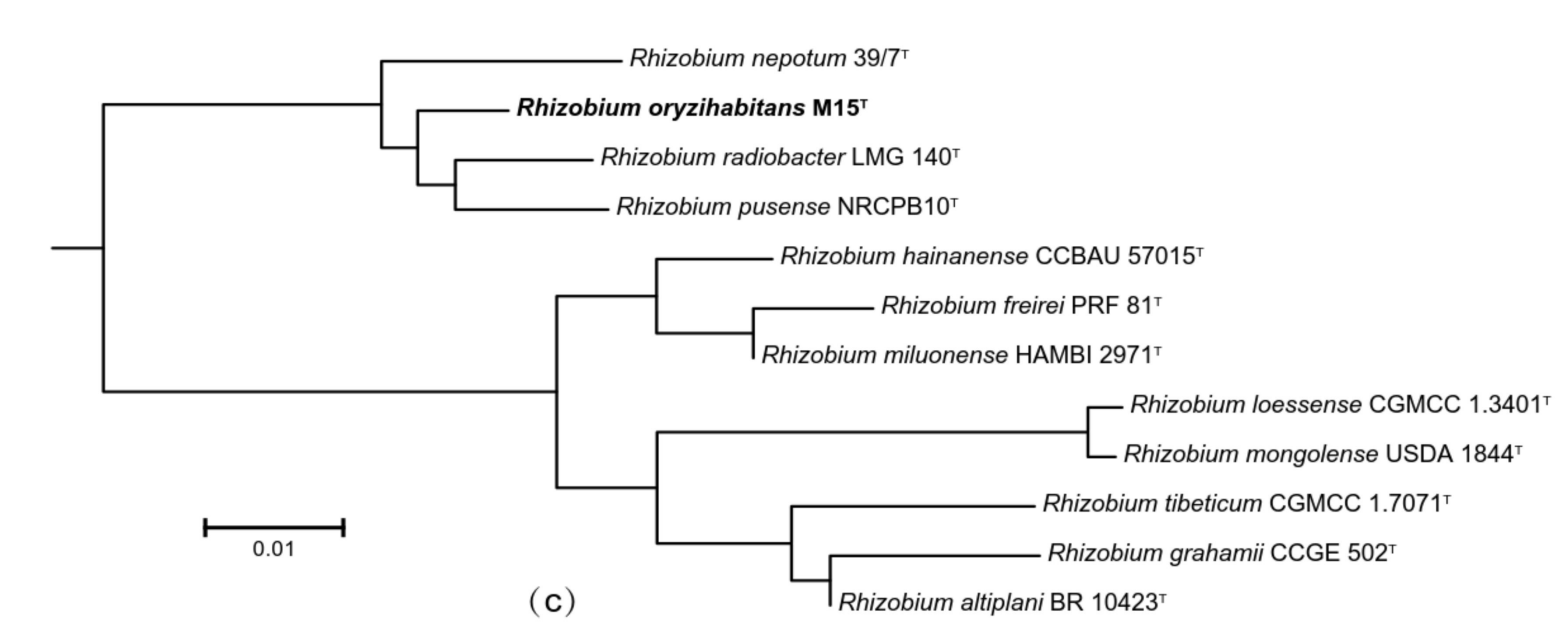
The comparative genome analysis of 12 closely related *Rhizobium* genomes. (**a**) The phylogenetic tree with the insert genome into species. (**b**) Genomic diversity numerically showing the homologous and non-homologous genes. Each strain was represented by an oval. Shared genes and species–specific genes were shown in center circle and petals, respectively. The strain name was located beside the oval. (**c**) Core genome-based phylogeny based on 2006 core orthologous proteins of strain M15^T^ and closely related species of genus *Rhizobium*.

Pan–genomic analysis of the 12 *Rhizobium* genomes revealed that 10,182 ortholog clusters that constituted the pan-genomes. The numbers of core genomes, strain-specific genomes and accessory genomes were 2006, 8082 and 7994, respectively (Figure 3A). The number of strain–specific genes in the 12 *Rhizobium* species was shown in Figure 3B. The phylogenomic analysis also supported the position of the strain M15^T^ was closest to *R. radiobacter* LMG140^T^, *R. pusense* NRCPB10^T^ and *R. nepotum* 39/7^T^ (Figure 3C). The results of virulence factors analysis showed that strain M15^T^ is differ from *R. radiobacter* LMG140^T^ in absence of VirB/VirD4 type IV secretion system and translocated effector Beps and virB–homolog (vbh) type IV secretion system, which is reported as a major virulence determinant for causing the crown gall disease of *Rhizobium radiobacter*. Therefore, despite the highest similarity to the pathogenic *R. radiobacter*, we can exclude the pathogenicity of strain M15^T^.

### 3.6. Secondary Metabolite Clusters

**Figure 4:**
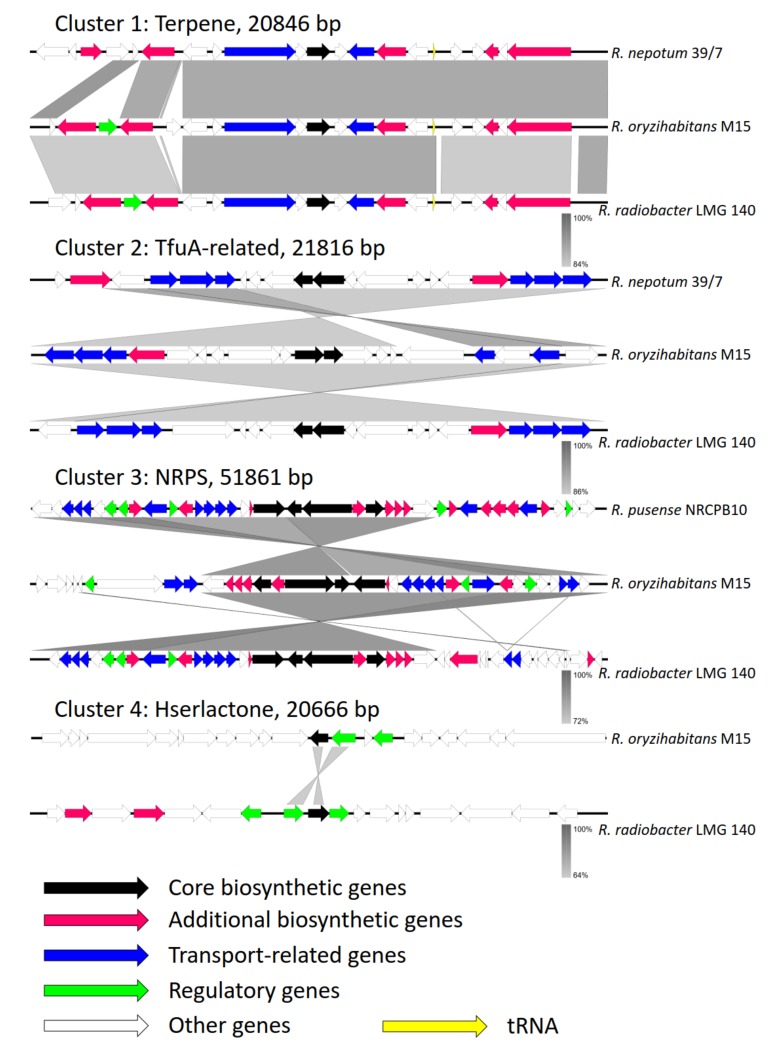
Comparison of biosynthetic gene clusters between strain M15^T^ and other closely related type species of the genus *Rhizobium*. Regions of conserved synteny were marked with grey (+) and green (−) shadow. Different genes are shown by different color arrows, and genes with the same color are homologous to each other.

Through the anti–SMASH genome analysis tool, four gene clusters of secondary metabolites have been identified in the genome of strain M15^T^, one terpene, one TfuA–related, one nonribosomal peptide synthetase (NRPS) and one hserlactone (Figure 4) [37]. Synteny and gene structure analysis of the four gene clusters were carried out depending on the homology and distribution of the genes in the gene clusters.

## 4. Conclusions

By means of biochemical, physiological and morphological characteristics, DNA–DNA hybridization and genotypic comparison of 16S rRNA, housekeeping genes and comparative genome analysis, strains M15^T^ are proposed to represent a novel species within the genus *Rhizobium* for which the name *Rhizobium oryzihabitans* sp. nov. is proposed. The description of *Rhizobium oryzihabitans* sp. nov. is summarized in Appendix A. Based on the analysis of plant growth-promoting agents, this novel isolate exhibited the capacity of producing siderophore, ACC deaminase, IAA and enhancing the rice plant growth. Thus, as PGPR, strain M15^T^ has the potential to be exploited as sustainable and environment-friendly inoculants in agriculture replacing fertilizers for enhancing plant growth.

## Patents

The work reported in the manuscript has been granted a Chinese invention patent (CN201810390383).

## Figures and Tables

**Table 1:** The phenotypic characteristics of strain M15^T^ and closely related strains. 1, M15^T^; 2, *R. radiobacter* LMG140^T^; 3, *R. pusense* NRCPB10^T^. All data are obtained from the present study. + (positive); w (weakly positive); − (negative).

Characteristics	1	2	3
*Growth in/at*			
NaCl (7.0%)	+	-	-
pH 11	+	-	+
*Carbon-source utilization*			
Dextrin	-	w	w
*N*-acetyl-D-galactosamine	+	+	-
Lactulose	w	+	+
D-psicose	-	+	+
Methyl pyruvate	-	+	w
Mono-methyl-succinate	-	+	w
Formic acid	-	w	+
*β*-hydroxy butyric acid	-	+	w
*α*-keto glutaric acid	+	+	-
L-alanine	+	+	w
L-threonine	-	w	w
Inosine	w	-	-
Uridine	w	-	-
2,3-Butanediol	-	-	w

**Table 2:** Cellular fatty acid contents of strain M15^T^ and the type strains of related *Rhizobium* type species. 1, M15^T^; 2, *R. radiobacter* LMG140^T^; 3, *R. pusense* NRCPB10^T^. All data were obtained in this study. Fatty acids representing < 1% in all strains were omitted. -, not detected.

Fatty Acid	1	2	3
C_9:0_	1.03	-	-
C_13:1_ at 12–13	3.2	1.75	1.98
C_16:0_	2.16	6.62	10.77
C_16:0_ 3-OH	1.88	5.38	5.47
C_19:0_ cyclo*ω8c*	3.13	38.23	40.77
Summed Feature 2 *	38.54	11.93	-
Summed Feature 3 *	1.65	1.03	2.22
Summed Feature 8 *	40.99	26.54	30.94

Note: * Summed features consist of two or more fatty acids that could not be separated by the Microbial Identification System. Summed feature 2 comprised aldehyde-C_12: 0_ and/or unknown equivalent chain length (ECL) 10.9525; summed feature 3 comprised C_16: 1_*ω7c* and/or C_16: 1_*ω6c*; summed feature 8 comprised C_18: 1_*ω7c* and/or C_18: 1_*ω6c*.

## Supplementary Materials

The following are available online at https://www.mdpi.com/2076-2607/8/4/608/s1.

## Appendix A

Description of *Rhizobium oryzihabitans* sp. nov.

*Rhizobium oryzihabitans* (L.n. oryza, rice; L. part. adj. habitans, inhabiting, dwelling; N.L. part. adj. oryzihabitans, rice–inhabiting).

Cells are Gram-negative, aerobic, flagellate and rods. Colonies are circular and cream-white on YMA at 30 ℃. Growth occurs from 15 ℃ to 50 ℃ and the pH range for growth is 5.0–12.0. The tolerant of NaCl concentrations is up to 7.0% (w/v). Positive reactions for nitrate reduction, aesculin hydrolysis, urease and nitro-D-methyl galactose activities, and assimilation of glucose, arabinose, mannose, mannitol, *N*-acetyl-glucosamine, maltose, gluconate, malic acid, citric acid, phenylacetic acid are positive. Catalase and oxidase positive. The following compounds utilize as carbon sources: *N*-acetyl-D-galactosamine, *N*-acetyl--D-glucosamine, adonitol, L-arabinose, D-arabitol, D-cellobiose, D-fructose, L-fucose, D-galactose, gentiobiose, *α*-D-glucose, m-inositol, *α*-D-lactose, lactulose, maltose, D-mannitol, D-mannose, D-melibiose, *β*-methyl-D-glucoside, D-raffinose, L-rhamnose, D-sorbitol, sucrose, D-trehalose, turanose, xylitol, acetic Acid, D-galactonic acid lactone, D-gluconic Acid, *α*-keto glutaric acid, DL-lactic acid, propionic acid, quinic acid, succinic acid, D-alanine, L-alanine, L-alanyl-glycine, L-asparagine, L-aspartic acid, L-glutamic acid, L-histidine, hydroxy-L-proline, L-ornithine, L-proline, L-pyroglutamic acid, L-serine, inosine, uridine, glycerol, glucose-1-phosphate, glucose-6-phosphate. The DNA G+C content of type strain is 59.28 mol %. Major cellular fatty acids are summed feature 8 (C_18:1_ *ω*7*c* and/or C_18:1_*ω*6*c*) and and Summed feature 2 (aldehyde-C_12:0_ and/or unknown equivalent chain length).

The type strain, M15^T^ (= JCM 32903^T^ = ACCC 60121^T^), was isolated from the root of rice.

The GenBank/The European Bioinformatics Institute EMBL-EBI (EMBL))/DNA Data Bank of Japan (DDBJ) accession numbers for the 16S rRNA, *recA*, *ropB* and *atpD* gene sequences of strain M15^T^ are MT023790, MT028481, MT028482 and MT028483, respectively. The complete genome have been deposited in GenBank under the accession numbers of SAMN14048699.

## References

[1] Frank B. (1889). Ueber die pilzsymbiose der *leguminosen*. Ber. Deutsch. Bot. Ges..

[2] Behrendt U., Kämpfer P., Glaeser S.P., Augustin J., Ulrich A. (2016). Characterization of the N_2_O-producing soil bacterium *Rhizobium azooxidifex* sp. nov.. Int. J. Syst. Evol. Microbiol..

[3] Gu T., Sun L.N., Zhang J., Sui X.H., Li S.P. (2014). *Rhizobium flavum* sp. nov., a triazophos-degrading bacterium isolated from soil under the long-term application of triazophos. Int. J. Syst. Evol. Microbiol..

[4] Zhang X., Li B., Wang H., Sui X., Ma X., Hong Q., Jiang R. (2012). *Rhizobium petrolearium s*p. nov., isolated from oil-contaminated soil. Int. J. Syst. Evol. Microbiol..

[5] Yoon J.H., Kang S.J., Yi H.S., Oh T.K., Ryu C.M. (2010). *Rhizobium soli* sp. nov., isolated from soil. Int. J. Syst. Evol. Microbiol..

[6] Sheu S.Y., Chen Z.H., Young C.C., Chen W.M. (2016). *Rhizobium ipomoeae* sp. nov., isolated from a water convolvulus field. Int. J. Syst. Evol. Microbiol..

[7] Liu Y., Wang R.P., Ren C., Lai Q.L., Zeng R.Y. (2015). *Rhizobium marinum* sp. nov., a malachite-green tolerant bacterium isolated from the sea water. Int. J. Syst. Evol. Microbiol..

[8] Rosenblueth M., Martinez-Romero E. (2004). *Rhizobium etli* maize populations and their competitiveness for root colonization. Arch. Microbiol..

[9] Zhao J.-J., Zhang X., Sun L., Zhang R.-J., Zhang C.-W., Yin H.-Q., Zhang X.-X. (2017). *Rhizobium oryziradicis* sp. nov. isolated from rice roots. Int. J. Syst. Evol. Microbiol..

[10] Zhang X., Sun L., Ma X., Sui X.H., Jiang R. (2011). *Rhizobium pseudoryzae* sp. nov. isolated from the rhizosphere of rice. Int. J. Syst. Evol. Microbiol..

[11] Zhang X.-X., Tang X., Sheirdil R.A., Sun L., Ma X.-T. (2014). *Rhizobium rhizoryzae* sp. nov. isolated from rice roots. Int. J. Syst. Evol. Microbiol..

[12] Zhang X.X., Gao J.S., Cao Y.H., Sheirdil R.A., Sheirdil X.C., Zhang L. (2015). Isolation and Proposal Novel Rice Promoting Endophytic Bacteria, *Rhizobium oryzicola* sp. nov. Int. J. Syst. Evol. Microbiol..

[13] Pravin V., Rosazlin A., Tumirah K., Ismail S., Boyce A.N. (2016). Role of Plant Growth Promoting Rhizobacteria in Agricultural Sustainability—A Review. Molecules.

[14] Poole P., Ramachandran V., Terpolilli J. (2018). Rhizobia: From saprophytes to endosymbionts. Nat. Rev. Microbiol..

[15] Ferreira C.M.H., Soares H.M.V., Soares E.V. (2019). Promising bacterial genera for agricultural practices: An insight on plant growth-promoting properties and microbial safety aspects. Sci. Total Environ..

[16] García-Fraile P., Carro L., Robledo M., Bahena M.H.R., Flores-Félix J.D., Fernández M.T., Mateos P., Rivas R., Igual J.M., Martínez-Molina E. (2012). *Rhizobium* promotes non-legumes growth and quality in several production steps: Towards a biofertilization of edible raw vegetables healthy for humans. PLoS ONE.

[17] Vincent J.M., Vincent J.M. (1970). The cultivation, isolation and maintenance of rhizobia. A Manual for the Practical Study of the Root-Nodule Bacteria.

[18] Lane D.J., Stackebrandt E., Goodfellow M. (1991). 16S/23S rRNA sequencing. Nucleic Acid Techniques in Bacterial Systematics.

[19] Martens M., Delaere M., Coopman R., De Vos P., Gillis M., Willems A. (2007). Multilocus sequence analysis of *Ensifer* and related taxa. Int. J. Syst. Evol. Microbiol..

[20] Martens M., Dawyndt P., Coopman R., Gillis M., De Vos P., Willems A. (2008). Advantages of multilocus sequence analysis for taxonomic studies: A case study using 10 housekeeping genes in the genus *Ensifer* (including former *Sinorhizobium*). Int. J. Syst. Evol. Microbiol..

[21] Kim O.S., Cho Y.J., Lee K., Yoon S.H., Kim M., Na H., Park S.C., Jeon Y.S., Lee J.H., Yi H. (2012). Introducing EzTaxon-e: A prokaryotic 16S rRNA gene sequence database with phylotypes that represent uncultured species. Int. J. Syst. Evol. Microbiol..

[22] Thompson J.D., Higgins D.G., Gibson T.J. (1994). CLUSTALW: Improving the sensitivity of progressive multiple sequence alignment through sequence weighting, position-specific gap penalties and weight matrix choice. Nucleic Acids Res..

[23] Kumar S., Stecher G., Tamura K. (2016). MEGA7: Molecular Evolutionary Genetics Analysis version 7.0 for bigger datasets. Mol. Biol. Evol..

[24] Saitou N., Nei M. (1987). The neighbor-joining method: A new method for reconstructing phylogenetic trees. Mol. Boil. Evol..

[25] Felsenstein J. (1981). Evolutionary trees from DNA sequences: A maximum likelihood approach. J. Mol. Evol..

[26] Tritt A., Eisen J.A., Facciotti M.T., Darling A.E. (2012). An Integrated Pipeline for de Novo Assembly of Microbial Genomes. PLoS ONE.

[27] Bankevich A., Nurk S., Antipov D., Gurevich A.A., Dvorkin M., Kulikov A.S., Lesin V.M., Nikolenko S.I., Pham S., Prjibelski A.D. (2012). SPAdes: A New Genome Assembly Algorithm and Its Applications to Single-Cell Sequencing. J. Comput. Biol..

[28] Chin C.-S., Peluso P., Sedlazeck F.J., Nattestad M., Concepcion G.T., Clum A., Dunn C., Omalley R., Figueroa-Balderas R., Morales-Cruz A. (2016). Phased diploid genome assembly with single-molecule real-time sequencing. Nat. Methods.

[29] Sergey K., Walenz B.P., Berlin K., Miller J.R., Bergman N.H., Phillippy A.M. (2012). Canu: Scalable and accurate long-read assembly via adaptive k-mer weighting and repeat separation. Genome Res..

[30] Meier-Kolthoff J.P., Auch A.F., Klenk H.P., Göker M. (2012). Genome sequence-based species delimitation with confidence intervals and improved distance functions. BMC Bioinform..

[31] Sasser M. (1990). Identification of Bacteria by Gas Chromatography of Cellular Fatty Acids.

[32] Glickmann E., Dessaux Y.A. (1995). Critical examination of the specificity of the Salkowski reagent for indolic compounds produced by phytopathogenic bacteria. Appl. Environ. Microbiol..

[33] Schwyn B., Neilands J.B. (1987). Universal chemical assay for detection and determination of siderophore. Anal. Biochem..

[34] Penrose D.M., Glick B.R. (2003). Methods for isolating and characterizing ACC deaminase-containing plant growth-promoting rhizobacteria. Physiol. Plant..

[35] Habibi S., Djedidi S., Ohkama-Ohtsu N., Sarhadi W.A., Kojima K., Rallos R.V., Ramirez M.D.A., Yamaya H., Sekimoto H., Yokoyama T. (2019). Isolation and Screening of Indigenous Plant Growth-promoting Rhizobacteria from Different Rice Cultivars in Afghanistan Soils. Microbes Environ..

[36] Arkin A.P. (2018). KBase: The United States department of energy systems biology knowledgebase. Nat. Biotechnol..

[37] Price M.N., Dehal P.S., Arkin A.P. (2010). FastTree 2-approximately maximum-likelihood trees for large alignments. PLoS ONE.

[38] Barton I.S., Fuqua C., Platt T.G. (2018). Ecological and evolutionary dynamics of a model facultative pathogen: *Agrobacterium* and crown gall disease of plants. Environ Microbiol..

[39] Blin K., Shaw S., Steinke K., Villebro R., Ziemert N., Lee S.Y., Medema M.H., Weber T. (2019). antiSMASH 5.0: Updates to the secondary metabolite genome mining pipeline. Nucleic Acids Res..

[40] Sullivan M.J., Petty N.K., Beatson S.A. (2011). Easyfig: A genome comparison visualizer. Bioinformatics.

[41] Jordan D.C., Genus I., Krieg N.R., Holt J.G. (1984). Rhizobium Frank 1889, 338AL. Bergey’s Manual of Systematic Bacteriology.

[42] Kim M., Oh H.S., Park S.C., Chun J. (2014). Towards a taxonomic coherence between average nucleotide identity and 16S rRNA gene sequence similarity for species demarcation of prokaryotes. Int. J. Syst. Evol. Microbiol..

[43] Richter M., Rosselló-Móra R. (2009). Shifting the genomic gold standard for the prokaryotic species definition. Proc. Natl. Acad. Sci. USA.

[44] Graham P.H., Sadowsky M.J., Keyser H.H., Barnet Y.M., Bradley R.S. (1991). Proposed minimal standards for the description of new genera and species of root- and stem-nodulating bacteria. Int. J. Syst. Bacteriol..

[45] Wayne L.G., Brenner D.J., Colwell R.R., Grimont P.A.D., Kandler O. (1987). International Committee on Systematic Bacteriology. Report of the ad hoc committee on reconciliation of approaches to bacterial systematics. Int. J. Syst. Bacteriol..

[46] Tighe S.W., De Lajudie P., DiPietro K., Lindström K., Nick G., Jarvis B.D. (2000). Analysis of cellular fatty acids and phenotypic relationships of *Agrobacterium*, *Bradyrhizobium*, *Mesorhizobium*, *Rhizobium* and *Sinorhizobium* species using the Sherlock Microbial Identification System. Int. J. Syst. Evol. Microbiol..

[47] Fu S.F., Wei J.Y., Chen H.W., Liu Y.Y., Lu H.Y., Chou J.Y. (2015). Indole-3-acetic acid: A widespread physiological code in interactions of fungi with other organisms. Plant Signal. Behav..

[48] Marques A.P.G.C., Pires C., Moreira H., Ragel A.O.S.S., Castro P.M.L. (2010). Assessment of the plant growth promotion abilities of six bacterial isolates using Zea mays as indicator plant. Soil Biol. Biochem..

[49] Ali S., Kim W.C. (2018). Plant Growth Promotion under Water: Decrease of Waterlogging-Induced ACC and Ethylene Levels by ACC Deaminase-Producing Bacteria. Front Microbiol..

[50] Tavares M.J., Nascimento F.X., Glick B.R., Rossi M.J. (2018). The expression of an exogenous ACC deaminase by the endophyte *Serratia grimesii* BXF1 promotes the early nodulation and growth of common bean. Lett. Appl. Microbiol..

