# Supplementary material for: Isolation, Identification and Characterization of Endophytic Bacterium Rhizobium oryzihabitans sp. nov., from Rice Root with Biotechnological Potential in Agriculture
Source: Microorganisms. 2020 Apr 22;8(4):608. doi: 10.3390/microorganisms8040608 (PMC7232506; doi:10.3390/microorganisms8040608)
Supplement: Supplementary file 1 [file microorganisms-08-00608-s001.zip › microorganisms-759964-supplementary.docx]

**Isolation, Identification and Characterization of**

**[Plant Growth-Promoting](https://www.frontiersin.org/articles/10.3389/fpls.2018.01473/full)****Endophytic Bacterium *Rhizobium oryzihabitans* sp. nov., from Rice Root with Biotechnological Potential in Agriculture**

**Juanjuan Zhao**^1^**, Xia Zhao**^2^**, Junru Wang**^1^**, Qi Gong**^1^**, Xiaoxia Zhang**^1^ **and Guishan Zhang**^1^**^*^**

^1^Key Laboratory of Microbial Resources Collection and Preservation, Ministry of Agriculture, Institute of Agricultural Resources and Regional Planning, Chinese Academy of Agricultural Sciences, Beijing 100081, P.R China.

^2^Beijing Research Institute of Chemical Engineering and Metallurgy, Beijing 101149, P.R China.

***Corresponding author:**

**Guishan Zhang**

Tel: +86-10-82108634; E-mail: [zhangguishan@caas.cn](mailto:zhangguishan@caas.cn), [gszhang86@gmail.com](mailto:gszhang86@gmail.com)

**Running title**: Isolation, Identification and Characterization of [Plant Growth-Promoting](https://www.frontiersin.org/articles/10.3389/fpls.2018.01473/full)Endophytic Bacterium *Rhizobium oryzihabitans* sp. nov.

**Subject category:** New taxa - *Rhizobiaceae*





**Figure S1.** Transmission electron microscopes image of the strain M15^T^.

***Rhizobium oryzihabitans* M15^T^(MT028481, MT028482, MT028483)**

*Rhizobium radiobacter* LMG140^T^(AM182121, AM295393, AM418785)

*Rhizobium pusense* NRCPB10^T^(HQ166059, FR871204, HQ114263)

*Rhizobium huautlense* HAMBI2409^T^(KF206825, KF206912, KF206569)

*Rhizobium miluonense* HAMBI2971^T^(KF206858, KF206945, KF206602)

*Rhizobium leguminosarum* LMG14904^T^(AM182125, AM295352, AM418783)

*Rhizobium undicola* LMG11875^T^(EF457952, EF457943, AM418784)

*Rhizobium phaseoli* ATCC14482^T^(EF113136, HQ670651, EF113151)

*Rhizobium lusitanum* p1-7^T^(DQ431674, JF318218, DQ431671)

*Rhizobium multihospitium* CCBAU83401^T^(EF490029, JF318220, EF490019)

*Rhizobium hainanense* CCBAU57015^T^(HM047132, JQ340071, GU726293)

56

100

98

98

100

98

53

100

0.05

**Figure S2.** Neighbour-joining phylogenetic tree showing the relationship between strain M15^T^ and the type strains of related species based on the housekeeping *recA*, *ropB*, *atpD* gene sequences. Bootstrap values with more than 50 % are shown on the nodes as percentages of 1000 replicates. *Rhizobium undicola* LMG11875^T^ was used as an outgroup. The scale bar equals 0.05 change per nucleotide position.

**Table S1**. The Average Nucleotide Identity values (%) based on BLAST (ANIb) for the genomes of the strain M15^T^ and the type species of the closely related *Rhizobium* spp..

| ANIb | *R. oryzihabitans* M15^T^ | *R. nepotum* 39/7^T^ | R. radiobacter LMG140^T^ | *R. pusense* NRCPB10^T^ | *R. freirei* PRF81^T^ | *R. hainanense* CCBAU57015^T^ | *R. miluonense* HAMBI2971^T^ | *R. mongolense* USDA1844^T^ | *R. altiplani* BR10423^T^ | *R. tibeticum* CGMCC1.7071^T^ | *R. loessense* CGMCC1.3401^T^ |
| --- | --- | --- | --- | --- | --- | --- | --- | --- | --- | --- | --- |
| *R. oryzihabitans* M15^T^  (SAMN14048699) | 100.00 | 88.80 | 88.11 | 86.00 | 75.11 | 74.76 | 74.68 | 74.54 | 74.34 | 74.31 | 74.27 |
| *R. nepotum* 39/7^T^  (GCA_000949865) | 88.80 | 100.00 | 87.45 | 85.42 | 74.76 | 74.77 | 74.75 | 74.60 | 74.26 | 74.11 | 74.62 |
| *R. radiobacter* LMG140^T^  (GCA_002008215) | 88.11 | 87.45 | 100.00 | 87.69 | 74.87 | 74.84 | 74.77 | 74.46 | 74.38 | 74.32 | 74.51 |
| *R. pusense* NRCPB10^T^  (GCA_002008275) | 86.00 | 85.42 | 87.69 | 100.00 | 74.74 | 74.49 | 74.32 | 74.51 | 74.15 | 73.98 | 74.30 |
| *R. freirei* PRF81^T^  (GCA_000359745) | 75.11 | 74.76 | 74.87 | 74.74 | 100.00 | 85.37 | 91.05 | 77.06 | 76.91 | 76.76 | 76.73 |
| *R. hainanense* CCBAU57015^T^  (GCA_900094555) | 74.76 | 74.77 | 74.84 | 74.49 | 85.37 | 100.00 | 85.34 | 76.91 | 76.54 | 76.36 | 76.69 |
| *R. miluonense* HAMBI2971^T^  ( jgi.1052910) | 74.68 | 74.75 | 74.77 | 74.32 | 91.05 | 85.34 | 100.00 | 77.03 | 76.77 | 76.54 | 76.76 |
| *R. mongolense* USDA1844^T^  (GCA_007827505) | 74.54 | 74.60 | 74.46 | 74.51 | 77.06 | 76.91 | 77.03 | 100.00 | 78.99 | 80.43 | 96.14 |
| *R. altiplani* BR10423^T^  ( GCA_001542405) | 74.34 | 74.26 | 74.38 | 74.15 | 76.91 | 76.54 | 76.77 | 78.99 | 100.00 | 86.13 | 78.93 |
| *R. tibeticum* CGMCC1.7071^T^  (GCA_900108425) | 74.31 | 74.11 | 74.32 | 73.98 | 76.76 | 76.36 | 76.54 | 80.43 | 86.13 | 100.00 | 79.27 |
| *R. loessense* CGMCC1.3401^T^  (jgi.1041465) | 74.27 | 74.62 | 74.51 | 74.30 | 76.73 | 76.69 | 76.76 | 96.14 | 78.93 | 79.27 | 100.00 |

**Table S2.** The DNA-DNA hybridization (DDH) values (%) for the genome of strain M15^T^ and the type species of the closely related *Rhizobium* spp..

| DDH | *R. oryzihabitans* M15^T^ | R. radiobacter LMG140^T^ | *R. pusense* NRCPB10^T^ | *R. nepotum* 39/7^T^ | *R. freirei* PRF81^T^ | *R. hainanense* CCBAU57015^T^ | *R. miluonense* HAMBI2971^T^ | *R. loessense* CGMCC1.3401^T^ | *R. altiplani* BR10423^T^ | *R. tibeticum* CGMCC1.7071^T^ | *R. mongolense* USDA1844^T^ |
| --- | --- | --- | --- | --- | --- | --- | --- | --- | --- | --- | --- |
| *R. oryzihabitans* M15^T^  (SAMN14048699) | 100.00 | 54.80 | 53.00 | 49.80 | 15.50 | 15.20 | 14.90 | 14.70 | 14.60 | 14.50 | 14.50 |
| *R. radiobacter* LMG140^T^  (GCA_002008215) | 54.80 | 100.00 | 64.60 | 54.90 | 15.30 | 15.50 | 15.00 | 15.10 | 14.70 | 14.80 | 14.80 |
| *R. pusense* NRCPB10^T^  (GCA_002008275) | 53.00 | 64.60 | 100.00 | 54.90 | 15.00 | 14.70 | 14.70 | 14.50 | 14.40 | 14.30 | 14.50 |
| *R. nepotum* 39/7^T^  (GCA_000949865) | 49.80 | 54.90 | 54.90 | 100.00 | 15.40 | 15.20 | 15.00 | 15.00 | 14.40 | 14.60 | 14.70 |
| *R. freirei* PRF81^T^  (GCA_000359745) | 15.50 | 15.30 | 15.00 | 15.40 | 100.00 | 42.50 | 60.70 | 17.40 | 17.60 | 17.20 | 17.60 |
| *R. hainanense* CCBAU57015^T^  (GCA_900094555) | 15.20 | 15.50 | 14.70 | 15.20 | 42.50 | 100.00 | 45.10 | 17.70 | 16.90 | 16.70 | 17.50 |
| *R. miluonense* HAMBI2971^T^  ( jgi.1052910) | 14.90 | 15.00 | 14.70 | 15.00 | 60.70 | 45.10 | 100.00 | 17.00 | 17.00 | 17.00 | 17.10 |
| *R. loessense* CGMCC1.3401^T^  (jgi.1041465) | 14.70 | 15.10 | 14.50 | 15.00 | 17.40 | 17.70 | 17.00 | 100.00 | 21.30 | 23.30 | 63.30 |
| *R. altiplani* BR10423^T^  ( GCA_001542405) | 14.60 | 14.70 | 14.40 | 14.40 | 17.60 | 16.90 | 17.00 | 21.30 | 100.00 | 36.40 | 21.30 |
| *R. tibeticum* CGMCC1.7071^T^  (GCA_900108425) | 14.50 | 14.80 | 14.30 | 14.60 | 17.20 | 16.70 | 17.00 | 23.30 | 36.40 | 100.00 | 24.40 |
| *R. mongolense* USDA1844^T^  (GCA_007827505) | 14.50 | 14.80 | 14.50 | 14.70 | 17.60 | 17.50 | 17.10 | 63.30 | 21.30 | 24.40 | 100.00 |
